# Importance of neutral processes varies in time and space: Evidence from dryland stream ecosystems

**DOI:** 10.1371/journal.pone.0176949

**Published:** 2017-05-09

**Authors:** Xiaoli Dong, David A. Lytle, Julian D. Olden, Tiffany A. Schriever, Rachata Muneepeerakul

**Affiliations:** 1 School of Life Sciences, Arizona State University, Tempe, Arizona, United States of America; 2 Nicholas School of the Environment, Duke University, Durham, North Carolina, United States of America; 3 Department of Integrative Biology, Oregon State University, Corvallis, Oregon, United States of America; 4 School of Aquatic and Fishery Sciences, University of Washington, Seattle, Washington, United States of America; 5 Department of Biological Sciences, Western Michigan University, Kalamazoo, Michigan, United States of America; 6 Institute of the Environment& Sustainability, Western Michigan University, Kalamazoo, Michigan, United States of America; 7 Department of Agricultural and Biological Engineering, University of Florida, Gainesville, Florida, United States of America; Aberystwyth University, UNITED KINGDOM

## Abstract

Many ecosystems experience strong temporal variability in environmental conditions; yet, a clear picture of how niche and neutral processes operate to determine community assembly in temporally variable systems remains elusive. In this study, we constructed neutral metacommunity models to assess the relative importance of neutral processes in a spatially and temporally variable ecosystem. We analyzed macroinvertebrate community data spanning multiple seasons and years from 20 sites in a Sonoran Desert river network in Arizona. The model goodness-of-fit was used to infer the importance of neutral processes. Averaging over eight stream flow conditions across three years, we found that neutral processes were more important in perennial streams than in non-perennial streams (intermittent and ephemeral streams). Averaging across perennial and non-perennial streams, we found that neutral processes were more important during very high flow and in low flow periods; whereas, at very low flows, the relative importance of neutral processes varied greatly. These findings were robust to the choice of model parameter values. Our study suggested that the net effect of disturbance on the relative importance of niche and neutral processes in community assembly varies non-monotonically with the severity of disturbance. In contrast to the prevailing view that disturbance promotes niche processes, we found that neutral processes could become more important when the severity of disturbance is beyond a certain threshold such that all organisms are adversely affected regardless of their biological traits and strategies.

## Introduction

Understanding community assembly—the processes responsible for observed spatiotemporal patterns of biodiversity—is a long-standing challenge in community ecology [1]. In recent years, a rich body of literature exploring the relative importance of niche *vs*. neutral processes has often resulted in polarizing outcomes [2–3]. According to the niche perspective, all species differ from each other, and their distribution and abundance are limited by environmental factors that select for particular biological traits expressed by species in the regional species pool [4]. The neutral perspective, in contrast, operates on the assumption that these interspecific differences are immaterial for explaining certain biodiversity patterns [5].

Both neutral and niche processes are important in community assembly; the challenge is to understand where, when, and how each affects community structure [6]. Now, it is also well recognized that environmental disturbance is an important force driving community structure and dynamics [7–9]. Both experimental evidence and theoretical models have suggested that disturbance can influence community assembly [10–13], including the relative importance of niche and neutral processes. The exact mechanisms by which the influence of disturbance operates, however, remains unresolved. Several studies, for example, support the hypothesis that neutral processes dominate in places characterized by more benign environments, whereas in harsher environments niche selection plays a more predominant role by filtering out species lacking disturbance resistance traits [14–17]. Other studies, however, suggest a more complex, non-monotonic relationship. In Sweden, Lepori & Malmqvist [11] reported that the strength of niche processes shaping macroinvertebrate communities increased with the severity of flood disturbance initially, but weakened once flood severity exceeded intermediate levels. These findings lend support to the notion that neutral processes may be important under severe disturbances, presumably because these disturbances promote random extinction and recolonization even for those organisms most resistant to disturbance. In short, considerable uncertainty regarding the roles of niche and neutral processes in community assembly remains.

Dryland streams are known for extreme hydrologic variability, where aquatic organisms face both severe drying events and massive flooding [18]. Hydrologic regimes are also heterogeneous across the landscape, consisting of a mosaic of perennial, intermittent, and ephemeral streams that vary greatly in flow permanence over the year [19]. Although all of these stream types experience flood disturbance, aquatic organisms must also cope with drought conditions in intermittent and ephemeral streams (hereafter “non-perennial”) that seasonally dry. For these reasons, dryland streams are an appropriate study system to evaluate the effects of disturbance on the relative strength of niche and neutral processes in community assembly. Indeed, a considerable amount of literature has shown that aquatic invertebrate assemblages in dryland streams are strongly influenced by hydrological variability in space and time [20–22]. In these systems, support for the role of niche processes (environmental filtering imposed by hydrological regime) has been well documented (e.g., [22–25]), yet the potential role of neutral processes and whether it might vary in space (i.e., different degrees of harshness: perennial vs. non-perennial streams) and time (i.e., different sequences of drying and flooding) has been notably overlooked.

The present study aims to address this knowledge gap by answering the following questions. First, are neutral processes more important for shaping community assembly in perennial versus non-perennial streams? Second, does hydrologic variability related to differing intensities of disturbance (i.e., drying and flooding) influence the contribution of neutral processes to community assembly? We analyzed macroinvertebrate community data spanning multiple seasons and years from 20 locations across a Sonoran Desert river network in Arizona, U.S.A., and built spatially- and temporally-explicit neutral metacommunity models to assess the changes of relative importance of neutral processes in space and in time.

## Methods

**Statement:** No specific permissions were required for accessing the study sites, and our study did not involve endangered or protected species.

### Overview

We incorporated temporal variability at two time scales (intra- and inter-annual) in spatially explicit neutral metacommunity models and analyzed the model performance to determine the relative importance of neutral processes. The commonly used approach to infer the relative importance of niche and neutral processes in different environments (e.g., harsh vs. benign environment, before vs. after disturbance) is based on β diversity (e.g., [11,17, 26–28]). In this case, stochastic neutral processes are implicated when the observed site-to-site similarity (β diversity) is not significantly different from expectations according to the null model—a purely random model of dispersal assembly alone. By contrast, the importance of niche processes is supported if the observed β diversity deviates significantly from null model prediction—i.e., significantly higher among-site species similarity than null model results. The null model approach, although designed to avoid bias caused by variation in α diversity, can still be affected by variation in α diversity [29]. This means that deviation from null model β diversity is not necessarily caused by niche processes, but could also be caused by difference in the variation of α diversity in the observed metacommunity and the metacommunity in the null model. Therefore, the current framework for using β diversity to differentiate between niche and neutral mechanisms is not robust [30]. Here we used a more intuitive method, which involved fitting the data to a neutral metacommunity model, and then calculating the model goodness of fit. We interpreted the model goodness-of-fit as the explanatory power, and thus relative importance, of neutral processes (as in [13]). For example, if the model goodness-of-fit is stronger for perennial versus non-perennial streams, then the relative importance of neutral processes is considered to be greater in perennial streams.

### Site and climate description

**Fig 1:**
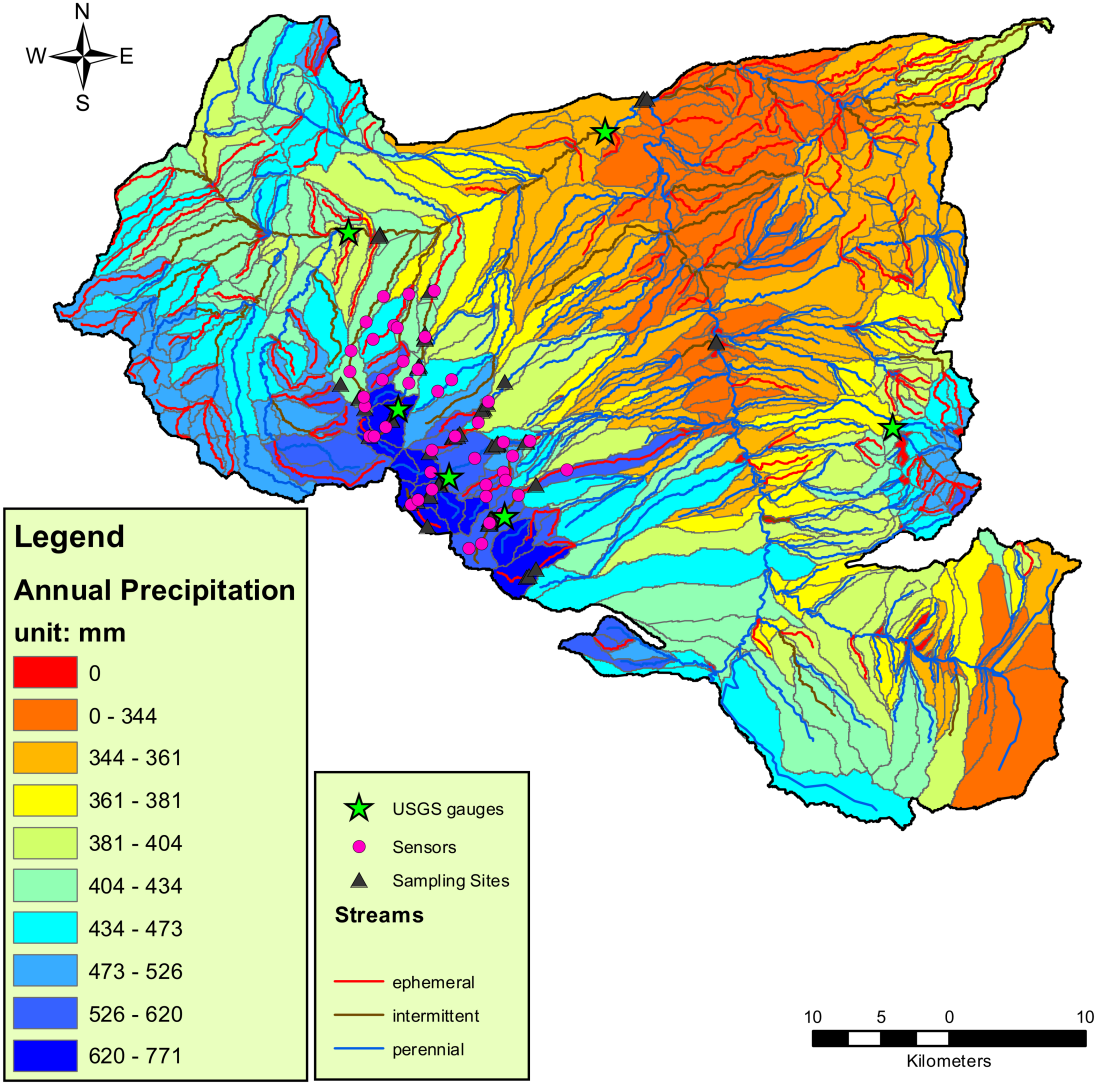
Map of Huachuca Mountains and San Pedro watershed, including streams labeled according to modeled hydrologic classifications (perennial, intermittent, and ephemeral), annual precipitation, invertebrate sampling points, USGS flow gauges (from north to south on the map: STAID 09471400, 09471380, 09471310, 09470800, 09470750, and 09470700), and electrical resistant sensors for recording water permanence. The main stem in the center of the map is the San Pedro River, and to the left are the Huachuca Mountains.

The study region included headwater streams of the San Pedro River located in the Sonoran Desert of southeastern Arizona, U.S.A. (Fig 1; 31° 29' 20.346'' N, 110° 24' 29.2932'' W). Precipitation in the region is highly variable from year to year, with approximately 60% of total annual rainfall occurring during intense summer (July-September) monsoon storms and the rest being delivering during more prolonged, moderate-intensity winter (December-April) storms.

**Fig 2:**
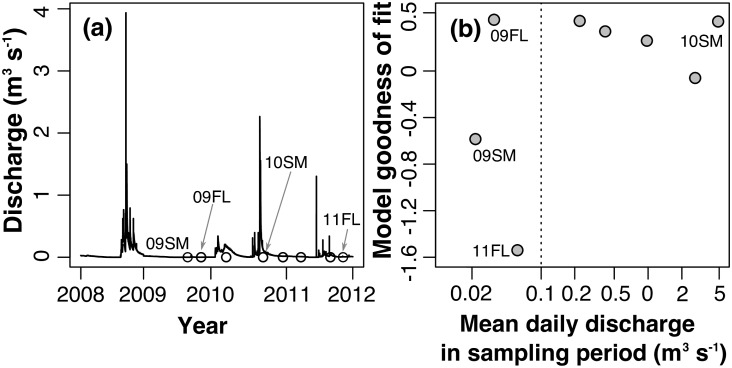
The effect of hydrology on the model performance. (a) Instantaneous discharge from Garden Canyon, AZ (USGS 09470800; Fig 1) between 2008 and 2011. Different gauges had different absolute discharge values, but showed similar hydrological patterns; the circle symbol indicates the time when sampling occurred; and (b) relationship of model fits (total model efficiency, *TE*, for α and β patterns combined) for those seasons *vs*. mean daily discharge during the sampling month, in the log scale, from gauges closest to the sampling sites. The shaded area denotes the high variability in model performance when mean daily discharge was very low.

Aquatic invertebrate communities were surveyed across eight sampling seasons, from 2009 to 2011, during the summer and winter high-flow periods and the fall low-flow period (Fig 2a). Mean discharge in 2010 was significantly higher than the other two years; the lowest mean discharge was observed in 2009. We collected invertebrate community samples from 20 sites in the Huachuca Mountains representing perennial, intermittent, and ephemeral streams (Fig 1). In each sampling season, a subset of all 20 sites, which had flowing water, were visited and sampled (S1 Table). Each site consisted of a 100-m-long stream reach in which all available microhabitat (primarily riffles and pools) were sampled with a D-net (500-μm mesh). In riffle sites, 0.33 m^2^ of stream substrate to a depth of 5 cm was disturbed, and invertebrate samples were collected immediately downstream. In pools, up to 6 m^2^ of pool area was swept with a D-net at an effort of 10 s m^-2^ pool habitat, with equal effort in benthic, pelagic, and edge habitats. The samples were preserved in 95% ethanol and were identified in the laboratory to the finest taxonomic level practical, usually to genus or species for insects and family or order for non-insects [23].

### River network characteristics and stream types

We used data from the National Hydrography Dataset Plus (NHDPlus) Version 2 (http://www.horizon-systems.com/NHDPlus/NHDPlusV2_home.php) to delineate the boundary of the San Pedro watershed and identify a total of 561 stream reaches (Fig 1). At higher elevations, streams are often fed by springs and can support perennial flow regimes. Further downstream, streams cross alluvial fans where surface water losses to evaporation and infiltration are high, and then become increasingly intermittent. Surface flow in these intermittent stream segments persists for weeks to months after precipitation. Further downstream, streams become ephemeral where the water table seldom rises above the streambed, and surface flow occurs for very brief periods (< 1 day) in response to extreme precipitation events. Below the alluvial fans, perennial rivers flow through fluvial floodplains. We classified each stream reach in the river network as ephemeral, intermittent or perennial according to a classification tree model (S1 Appendix) relating remotely-sensed physical data and measured water permanence from an array of field sensors (see [31]).

### Simulating inter- and intra- annual hydrological variability

Discharge data during the study period (2009–2011) was used to determine the duration of high flow and low flow periods in a year. There are six USGS gauges located within the study area (Fig 1). Based on the discharge regime from these gauges between 2009 and 2011, we estimated 16 weeks for the duration of the winter high flow period, 12 weeks for the summer high flow period, and 12 weeks each for spring and fall low flow periods in an average year.

We obtained the spatial gradient of annual precipitation across the watershed from USGS National Atlas GIS data (Fig 1). This represented the precipitation spatial heterogeneity in an average year (averaged over the period of 1961 to 1990). We extracted the precipitation for each catchment within the watershed. To simulate the inter-annual variability in precipitation, we obtained annual precipitation data (non-spatial) between 1922 and 2014 from NCEP North American Regional Reanalysis (NARR) by National Oceanic & Atmospheric Administration (NOAA) for southern Arizona. We used a first-order autoregressive model to fit this long-term dataset:

$$PT_{t}-\bar{PT}=\rho \left(PT_{t-1}-\bar{PT}\right)+Z_{t}$$ (1)

where *PT* was annual precipitation, *ρ* was the lag-1 autocorrelation coefficient, and *Z* was the stochastic term. We fitted *Z* with a gamma distribution with shape parameter *k* = 3.40, scale parameter *θ* = 4.95, and a shift of -16.86 (to keep *E*[*Z*_*t*_] = 0). This first-order autoregressive model is then used to simulate realistic sequences of annual precipitation, which preserve the observed mean, variance, and one-year-lag autocorrelation, experienced by organisms in the neutral metacommunity model (S1 Fig).

### Estimating habitat capacity

An important prerequisite for an effective neutral metacommunity model is a reasonable estimation of habitat capacity [32]. Here, we assumed that water availability is a proxy of habitat capacity (*HC*), which is the amount of space and resources available to support species occupancy at any particular site. We made use of the power-law relationship between α diversity (watershed scale) and *HC*, which is conceptually similar to the well-known species-area relationship (species richness increases with sample area; here we replaced sample area with *HC*). We used the product of precipitation and watershed area (*PT*×*WA*) to estimate *HC*. We observed that α diversity first increased with *PT*×*WA*, peaked at an intermediate value, and decreased at high values of *PT*×*WA* (S2 Appendix). Based on this empirical pattern, we estimated habitat capacity of each catchment (*HC*_*i*_) by its *PT*×*WA* via the following formula (derivation in S2 Appendix):

$$HC_{i}={\left(C{\left(PT_{i}\times WA_{i}\right)}^{0.9}exp\left(-PT_{i}\times WA_{i}/4700\right)\right)}^{a}$$ (2)

where *a* and *C* parameters were to be determined by a model fit. It is worth noting that because the estimation of the habitat capacities is imperfect, the error in such estimation could contribute to the error between the predicted and empirical biodiversity patterns. Therefore, the model goodness-of-fit may underestimate the importance of neutral processes. Nonetheless, the model fit is still a good metric for *relative* importance of neutral processes under different environmental settings.

### Description of neutral metacommunity models

We implemented a neutral model on the stream network consisting of 561 stream reaches (561 sites distributed across the entire watershed depicted in Fig 1), which are a mix of ephemeral, intermittent, and perennial streams, as described above. Aquatic invertebrates in these 561 streams form a metacommunity. We constructed a spatially explicit neutral metacommunity model that takes into account the intra- and inter-annual variability in hydrologic conditions.

The model was similar to the one developed by Muneepeerakul et al. (2008) [32], which included stochastic dispersal, reproduction, mortality, and speciation. The dispersal kernel was assumed to be the bivariate Students’ *t* or “2Dt” kernel [33], which can be written as

$$K_{ij}=C_{j}\frac{p}{\pi l_{0}^{2}{\left[1+{\left(\frac{L_{ij}}{l_{0}}\right)}^{2}\right]}^{p+1}}$$ (3)

where *K*_*ij*_ is the probability that an organism produced at site *j* arrives at site *i* after dispersal; *C*_*j*_ is a normalization constant to ensure that for every site *j*, Σ_*i*_ *K*_*ij*_ = 1, i.e., no organisms traveled out of the metacommunity. *L*_*ij*_ is the Euclidean distance between two habitats (our preliminary results on distance decay relationship as shown in S3 Appendix suggested that Euclidean distance is appropriate). This dispersal kernel was determined by *l*_*0*_ (scale parameter) and *p* (shape parameter): *l*_*0*_ is the distance over which, after dispersal, the ratio between its offspring and those at the origin location is 2^-(1+*p*)^ [34]. The 2Dt kernels were chosen because they can exhibit a wide range of behaviors, from the heavy-tailed Cauchy kernel when *p* approaches 0 to the thin-tailed Gaussian kernel when *p* approaches ∞ and others in between.

At each time step, a randomly selected individual died (with probability *m*) and the resources that previously sustained that individual were available to sustain a new individual. With probability *v*, the diversification rate, the vacancy was occupied by a new species (the diversification rate is a per-birth rate and is due to speciation or to immigration of a new species from outside the metacommunity). With probability 1-*v*, the vacancy was occupied by a species already existing in the metacommunity. In the latter case, the probability *P*_*ij*_ that the vacancy in site *i* would be colonized by a species from habitat *j* was determined as follows:

$$P_{ij}=\left(1-v\right)\frac{K_{ij}H_{j}}{\sum_{k=1}^{N}K_{ik}H_{k}}$$ (4)

where *K*_*ij*_ is the dispersal kernel, *H*_*k*_ is the habitat capacity of site *k*, and *N* is the total number of sites (i.e., communities). All the individuals arriving at site *j* had the same probability of colonizing the vacancy at site *i* where the death took place.

To simulate intra-annual variability (i.e., seasonality), the model incorporated varying habitat capacity and duration for each flow season. Perennial streams were assumed to have the same habitat capacity in all four seasons, equal to the value estimated by Eq (2). The habitat capacity for intermittent and ephemeral streams varied with seasons, modified by a season-specific weight (< 1). The values of the weights were estimated from the observed species abundance data for the corresponding stream types. When the habitat capacity increases from one flow period to the next, there will be empty sites available to be occupied in the reaches in that catchment. These unoccupied sites will be recolonized with a probability *r*. The recolonizing species can be a species already existing in the metacommunity with probability 1-*v*, or a new species with probability *v*. On the other hand, if the habitat capacity decreases from one flow period to the next, a randomly selected set of individuals die, with the number of deaths equal to the difference in habitat capacity between two flow periods (i.e., mimicking population losses resulting from disturbance).

The first-order autoregressive model was used to simulate changes in habitat capacity associated with inter-annual variability in precipitation (see Section “Simulating inter- and intra- annual hydrological variability”). From one year to the next, when mean precipitation changes, it proportionally changes the habitat capacity of each catchment. As with the transitions between seasons, if habitat capacity increases, unoccupied sites appear in the first time step of the year and are recolonized with a probability *r* in the following time steps. The recolonizing species can be a species already existing in the metacommunity with probability 1-*v*, or a new species, with probability *v*. If habitat capacity decreases from one year to the next, a randomly selected set of individuals die, the number dying equaling the difference in habitat capacity between the two years.

Each time step in the model represented one week, and the model required 80,000 time steps (about 1500 y in model time) to reach a statistical steady state, i.e., when the biodiversity patterns showed no directional trend in the mean local species richness or the total species richness. After the model reached steady state, an additional 1000 y of model time was processed to estimate average community conditions; biodiversity patterns derived from these steady-state conditions were compared to the observed empirical patterns.

### Quantifying model goodness-of-fit

We compared the fit between observed and modeled α and β diversity (measured by Chao similarity index [35]) to assess the performance of the model. The best-fit parameter set was chosen by the following procedure. We ran a number of simulations with different sets of parameters distributed over a wide range. For every simulation, we computed Nash-Sutcliffe model efficiency coefficient (*E*, for model efficiency) [36] for the two biodiversity patterns: α and β diversity. The Nash-Sutcliffe model efficiency coefficient is identical to the coefficient of determination (*R*^*2*^) in their formula [36]. The difference is that *R*^*2*^ is typically used as a measure of goodness-of-fit of statistical models, and Nash-Sutcliffe model coefficient is typically used to quantify how well a model simulation can predict the outcome, and is commonly used in assessing performance of hydrological models [36]. The model fit for pattern *k*, *E*_*k*_ (*k* = 1 for α diversity, 2 for β diversity), was estimated by one minus the mean squared deviation between data and predicated values normalized by the data variance; this can be expressed as follows:

$$E_{k}=1-\frac{\sum_{i=1}^{N_{k}}{\left(x_{k,i}-{\hat{x}}_{k,i}\right)}^{2}}{\sum_{i=1}^{N_{k}}{\left(x_{k,i}-\langle x_{k}\rangle \right)}^{2}}$$ (5)

where *N*_*k*_ was the number of data points used in fitting pattern *k*, *x*_*k*,*i*_ and ${\hat{x}}_{k,i}$ were data point *i* (data points from all eight sampling events across three years) of pattern *k* and its predicted value, respectively, and 〈*x*_*k*_〉 the mean value of the data points of pattern *k*. We then defined total model efficiency coefficient, *TE* of each parameter set as *E*_1_ + *E*_2_. *TE* was used as a metric to compare model goodness-of-fit, and the parameter set with maximum *TE* was selected as the best model. A simulation model may be calibrated, but the predicted values of the outcome variable ${\hat{x}}_{k,i}$ are not inferred from the observed values. Therefore, for a poorly performing model, the sum of squares of the model error, $\Sigma_{i=1}^{N_{k}}{\left(x_{k,i}-{\hat{x}}_{k,i}\right)}^{2}$, may be greater than the total sum of squares, $\Sigma_{i=1}^{N_{k}}{\left(x_{k,i}-\langle x_{k}\rangle \right)}^{2}$, resulting in negative *TE*. In this particular study, a negative *TE* indicates that non-neutral processes may be playing strong roles in shaping the community assembly.

To answer the first question of this study—are neutral processes more important in perennial or non-perennial streams?—we extracted the perennial stream subset and non-perennial stream subset (grouping intermittent and ephemeral streams), and compared the goodness-of-fit of the best model for the two stream types. To answer the second question—does flow variation influence their relative importance in community assembly, and how?—we calculated the best model’s goodness-of-fit for each sampling event, and examined the relationship between sampling event-specific model goodness-of-fit and the flow condition (mean daily discharge) of each sampling event.

## Results

Using the aggregated result from the best model (the model with the highest total model efficiency coefficient *TE*; best parameter set in S2 Table), we compared the model goodness-of-fit for two different stream types, i.e., perennial and non-perennial streams. Model performance was notably better in predicting biodiversity patterns for perennial streams than non-perennial streams (Table 1). Analysis of the empirical dataset (prior to modeling) showed that local species richness (α diversity) was consistently higher in perennial streams than that in non-perennial streams across all sampling seasons (S3 Table).

**Table 1:** Model performance (measured by total model efficiency coefficient) for perennial and non-perennial streams.

Stream type	α diversity	β diversity
**Perennial**	0.32	0.22
**Non-perennial**	-0.21	-0.36

**Fig 3:**
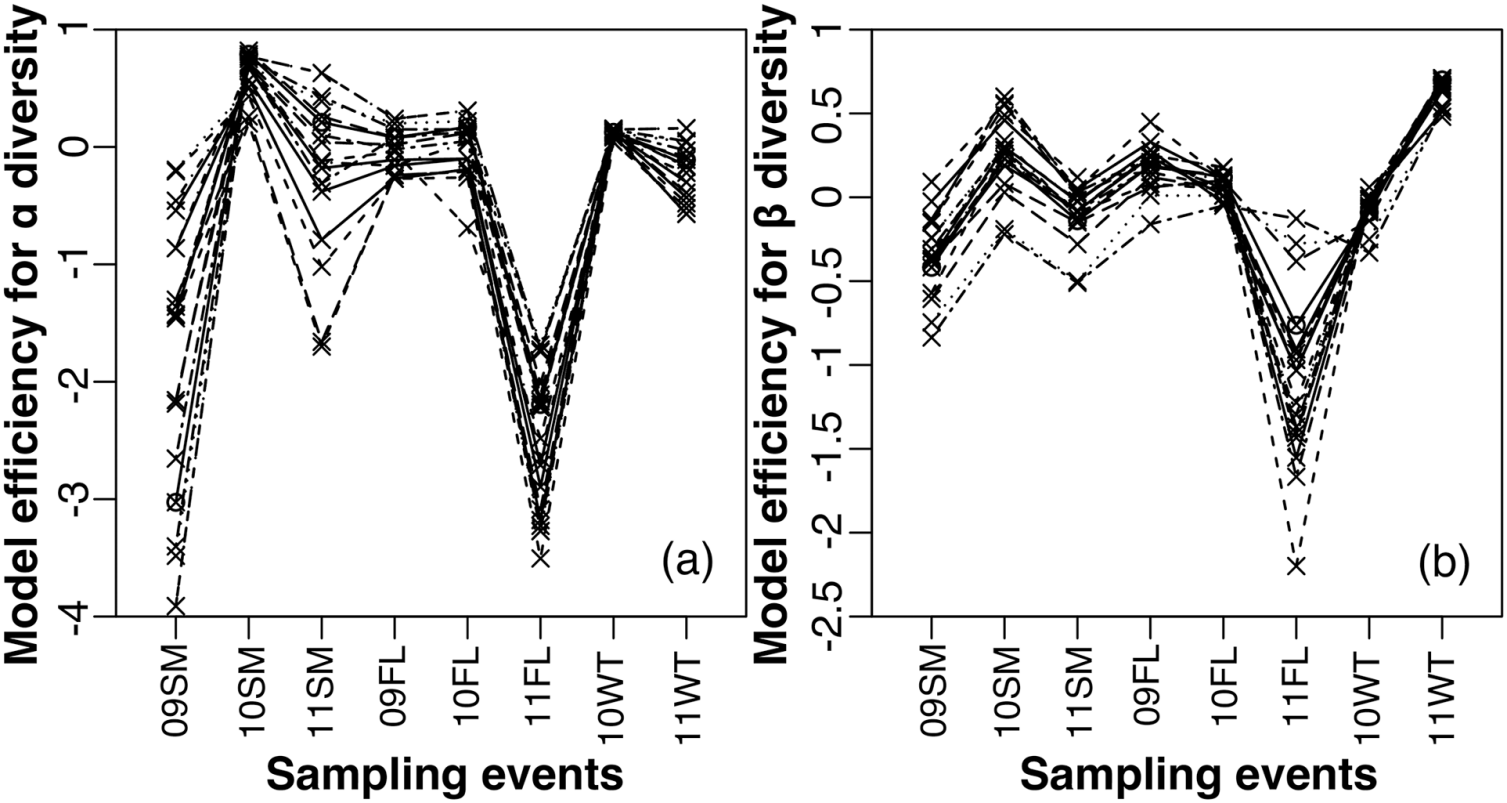
The model efficiency *E*_*k*_ of the model fitted with different parameter sets (*n* = 20, results from the same parameter set were connected with a line) within a range of values for (a) α diversity pattern, and (b) β diversity pattern across 8 sampling events in three years (2009, 2010, 2011). Season abbreviations are as follows: summer (‘*SM*’), fall (‘*FL*’), and winter (‘*WT*’).

We then checked the year- and season- specific result from the model. For α diversity, the best fit occurred in 2010 summer, the high flow period in a very wet year, and the poorest fit occurred in 2009 summer and 2011 fall (Table 2; Fig 3a). Model performance for β diversity was strongest for 2010 summer and 2011 winter and weakest for fall 2011 (Table 2; Fig 3b). Because of the high inter- and intra-annual hydrological variability, we used the mean daily stream discharge during the sampling period to quantify the “wetness” of each period in different seasons and years: the mean daily discharge from the USGS gauges close to sampling sites in each sampling event (Fig 1) during the sampling month. We found that when the mean daily discharge was very low, below ~ 0.1 m^3^ s^-1^, there was large variability in model performance, including even negative *TE* (Fig 2b). With greater mean daily discharge (above 0.1 m^3^ s^-1^), the model goodness of fit first decreased with discharge and then increased with discharge, with the poorest model performance at the intermediate range of discharge.

**Table 2:** Performance of the best-fit model (measured by model efficiency coefficient) for prediction of α diversity and β diversity (Chao similarity index) across the eight sampling seasons.

Metrics	Summer			Fall			Winter	
	2009	2010	2011	2009	2010	2011	2010	2011
**α diversity**	-0.98	0.49	0.44	0.46	0.47	-1.28	-0.12	0.30
**β diversity**	-0.19	0.36	0.08	0.42	0.21	-1.80	0.00	0.56

According to the best fit model, the overall explanatory power provided by neutral processes for α diversity was 36%, and for β diversity was 16% for this system (Table 3). When aggregated by year (method on aggregation described in S4 Appendix), the wettest year (2011) had the best fit for α diversity (Table 3). When aggregated by season, model performance for the fall season exceeded that for the summer or winter season for α and β diversity patterns (Table 3). Our findings on how model performances vary in different times and across stream types are robust to changes in parameter values (Fig 3). For both α and β diversity, the absolute model goodness-of-fit could be improved or worsened depending on the values of parameters, but relative differences among sampling periods in model goodness-of-fit remain unchanged (Fig 3).

**Table 3:** Model performance (measured by total model efficiency coefficient) for the best model. We aggregated season- and year-specific results from the model via averaging.

	Season-year-specific	Aggregated by season	Aggregated by year
**α diversity**	0.36	0.27	0.32
		*sm = -0.07	§*y09* = 0.33
		†*wt* = 0.14	||*y10* = 0.41
		‡*fl* = 0.36	¶*y11* = 0.43
**β diversity**	0.16	0.07	0.10
		*sm* = -0.05	*y09* = 0.20
		*wt* = 0.02	*y10* = 0.00
		*fl* = 0.20	*y11* = 0.20

*summer;

^†^winter;

^‡^fall;

^§^year 2009;

^||^year 2010;

^¶^year 2011

## Discussion

In this study, we developed a spatiotemporal neutral model for aquatic invertebrate communities in dryland streams to investigate whether and how the relative importance of neutral processes varies across space and time. Despite some non-ideal conditions for neutrality [37], namely relatively low richness compared to streams in other biomes [38] and generally strong dispersal limitation among freshwater invertebrates [39], dryland streams offer great natural laboratories for investigating the effects of temporal variability on the relative strength of niche and neutral processes in community assembly. This is because these streams are highly variable in time, strongly heterogeneous in space, and harbor ecological communities that have historically experienced such environmental variability. We used the goodness of fit of the neutral metacommunity model to evaluate the relative importance of neutral processes in perennial and non-perennial streams and under different hydrological conditions. Even though we only had eight sampling events—which limited the range of hydrological conditions represented and the strength of statistical inference—our results were informative. We found greater relative importance of neutral processes in perennial streams and during relatively low flow periods and periods of very high flow that represented times of hydrologic disturbance. However, when flow was very low (mean daily discharge < 0.1 m^3^ s^-1^), the model performance exhibited large variability, with *TE* ranging from -1.6 to 0.5 (Fig 2b); the negative *TE* values hinted at the possibility of strong non-neutral processes. These results suggested that the importance of neutral assembly processes vary both in space and time for the aquatic invertebrate community in this dryland stream network.

The neutral model provided consistently better model fits for invertebrate communities of perennial streams than for non-perennial streams: neutral processes explained 20%-30% of species assembly in perennial streams, but the low (or negative) *TE* indicated that neutral processes play much weaker roles in non-perennial streams (Table 1). Although both perennial and non-perennial streams experience disturbances in the form of floods, droughts in non-perennial streams are likely stronger disturbances for aquatic macroinvertebrates. Leigh & Datry [25] recently assessed the influence of drying on macroinvertebrate communities in Australia and Europe over broad spatial and temporal scales, and found that drying is more important to species diversity compared to other flow-related determinants. Fritz & Dodds [40] showed that stream macroinvertebrates are typically more resistant and resilient to flooding than to drying. In our own study system, Bogan & Lytle [21] monitored two stream pools for eight years, and found that invertebrate communities underwent a shift in species composition in response to a transition from perennial flow to intermittent. In this sense, perennial streams, despite their frequent flood disturbances, are relatively more benign habitats for macroinvertebrate communities than non-perennial streams that experience drying. This agrees with the theoretical prediction that neutral processes are more important in systems that have less environmental fluctuation (more benign habitats) [17]. In contrast, flow intermittency serves as a stronger environmental filter to select for species with biological traits and behavioral strategies to survive drying phases, leading to greater effects of niche processes and weaker roles of neutral processes in non-perennial.

Across the entire dryland stream network, the model performance varied with stream discharge in the sampling period: when mean daily discharge was greater than 0.1 m^3^ s^-1^, model goodness-of-fit decreased with discharge, and increased again when the discharge was very high (Fig 2b). A decrease in model performance with discharge indicated that neutral processes became less important as the stream became wetter (often associated with floods). This is consistent with the current understanding that as the habitat is more disturbed, niche processes become more important [14–17]. However, at the very high flow end (daily discharge at about 5 m^3^ s^-1^), with their large flood disturbances, neutral processes explained up to ~50% of the variance (Fig 2b). This is contradictory to the prevailing view that disturbances promote niche processes [14–17]. A potential explanation is that the severe disturbances cause random recolonization and extinction even among the regional taxa that are most resistant to disturbance [11]. The summer 2010 sampling took place two weeks after a 5-year flood (i.e., 20-percent annual exceedance probability) (Fig 2a). Faced with severe floods, the protection against flood scour provided by biological traits (e.g., streamlined body shape, small body size, and ability for attachment to the substratum [7]) is probably very limited, and community assembly may be driven more by neutral processes.

At the other end of hydrological spectrum—the very low flow conditions (mean discharge lower than 0.1 m^3^ s^-1^)—the roles of neutral processes in community assembly were mixed: ranging from moderate importance of neutral processes to likely dominance of non-neutral processes. The variable relative importance of neutrality could be because our measure of discharge was calculated for the entire sampling period, and thus did not isolate the flow conditions immediately prior to sampling nor capture the long-term flow patterns at the site. For example, although 2009 (summer and fall) and 2011 (fall) had similar low discharge during the sampling periods, the duration of low flow periods varied greatly: the very low flow period lasted for 15 months prior to 2009 fall sampling, 12 months prior to 2009 summer sampling, and just two months prior to 2011 fall sampling. Water-retaining refuges could provide effective protection against drought for most populations for a certain period of time; however, confronted with prolonged severe drought, these refuges would be compromised by reductions in size and worsening water quality [41]. These results suggest that when disturbances are sufficiently severe—large flood or prolonged and severe drought—the risk of mortality may be decoupled from the species’ traits and identity, resulting in more neutral process-dominated biodiversity patterns. Lepori & Malmqvist [11] showed similar results, where extreme disturbances triggered neutral processes for an aquatic invertebrate community in streams in North Sweden. There are also alternative explanations for greater importance of neutral processes during very high flow periods. For example, when stream flow is very low or very high, the whole landscape is more homogeneous (uniformly connected high stream flow), while intermediate and low flow promotes higher flow heterogeneity across landscapes by creating higher degrees of habitat patchiness. Theoretical models [37, 42] suggest that the validity of the neutrality assumptions increases as a more homogeneous environment enables higher niche overlap. This explanation is somewhat speculative at this point, as one needs quantification of landscape heterogeneity to support it—a worthwhile future research direction. Taken together, these findings suggest that the theoretical framework needs to be modified to recognize that the net effect of disturbance on the relative importance of community assembly forces could be non-monotonic and severity-dependent. Additionally, the relative contribution by neutral processes to community assembly could vary both in space and in time, as demonstrated in our study.

## Supporting information

S1 Appendix Methods for characterizing stream types. (DOCX)

S2 Appendix Derivation of the model to estimate habitat capacity. (DOCX)

S3 Appendix Methods for determining distance matrix for dispersal. (DOCX)

S4 Appendix Comparing model goodness-of-fit at different aggregate levels. (DOCX)

S1 Fig Observed hydrological regime of inter- and intra-annual variability and hydrological regime used in the model. (DOCX)

S1 Table Time of sampling across the 20 sites (streams) between 2009 and 2011. (DOCX)

S2 Table List of parameters in the model, their ecological interpretations, and their values used in the best-fit models. (DOCX)

S3 Table Mean local species richness (and the standard deviation) across sampled sites in each sampling season in perrenial streams and non-perrenial streams. (DOCX)
