# Supplementary material for: Importance of neutral processes varies in time and space: Evidence from dryland stream ecosystems
Source: PLoS One. 2017 May 9;12(5):e0176949. doi: 10.1371/journal.pone.0176949 (PMC5423606; doi:10.1371/journal.pone.0176949)
Supplement: S1 Appendix — (DOCX) [file pone.0176949.s001.docx]

**Appendix S1.** Methods for characterizing stream types

To identify the hydrological type for each stream reach in the river network, i.e., ephemeral, intermittent, and perennial or near-perennial streams, we constructed a classification tree model using field sensor data on water permanence from Jaeger & Olden (2012) [1]. We modeled three categories of flow permanence: 0 (ephemeral), 1 (intermittent), and 2 (perennial or near-perennial), as a function of watershed geology, upstream drainage area, and elevation. The geology data (i.e., the types of mineral) were collected from USGS (Arizona Geology layer: <http://mrdata.usgs.gov/geology/state/state.php?state=AZ>; and North American Geology layer for Mexico: <http://www.arcgis.com/home/item.html?id=2967ae2d1be14a8fbf5888b4ac75a01f>). The geology data layer was imposed on the catchment delineation layer so that each catchment in the river network was labeled with its major mineral type, which was used as one of the input variables for the classification tree model. The analysis was carried out in R [2] with the package ‘tree’ [3]. It is important to note that the sample size for the classification tree model was only 25 sensors, that is, flow permanence data for 25 streams (compared to a total of 561 streams within the whole drainage area) and sensors were concentrated within a relatively small area (sensors were located in streams sampled for macroinvertebrates) (Fig 1). While these sensors did not capture the entire spatial extent of variability of the large drainage areas, their recorded data ranged from 0 to 100%, representing the full gradient of flow permanence.

**References:**

1. Jaeger KL, Olden JD. Short communication electrical resistance sensor arrays as a means to quantify longitudinal connectivity of rivers*.* River Res Applic 2012;1852: 1843–1852.
2. R Core Team. R: A language and environment for statistical computing. R Foundation for statistical Computing, Vienna, Austria. 2014; ISBN 3-900051-07-0, URL <http://www.R-project.org/>
3. Ripley B. 2015. Package ‘tree’, version 1.0.35. http://cran.r-project.org/web/packages/tree/tree.pdf
