# Supplementary material for: Importance of neutral processes varies in time and space: Evidence from dryland stream ecosystems
Source: PLoS One. 2017 May 9;12(5):e0176949. doi: 10.1371/journal.pone.0176949 (PMC5423606; doi:10.1371/journal.pone.0176949)
Supplement: S2 Appendix — (DOCX) [file pone.0176949.s002.docx]

**Appendix S2.** Derivation of the model to estimate habitat capacity

The purpose of this sub-model is to use *PT*×*WA* to estimate habitat capacity, which is needed in the neutral metacommunity model. Here are the steps:

1. We found that α diversity first increased with *PT*×*WA*, then decreased, with a heavy tail (Fig. A). This suggests that we could use a transformed gamma distribution to fit the relationship between α diversity and *PT*×*WA.* The best fit gamma distribution is expresses as follows

$$\alpha\left( {PT}_{i}\times{WT}_{i} \right)=Gamma\left( 1.9, 4700 \right)\times9500 (Eq.1)$$

That is, at any site, the α diversity can be expressed by precipitation and watershed area of that site with a gamma distribution with shape parameter 1.9 and scale parameter 4700 (or rate parameter 1/4700), times a constant 9500. This is an empirical relationship.

1. Once we get this empirical relationship, for each site, with known *PT*×*WA*, we can get its α diversity.
2. However, we needed habitat capacity, not α diversity. To get habitat capacity, we used the species-area relationship (actually the “species-*habitat capacity*” relationship) to infer habitat capacity of each catchment (*HC_i_*) based on the estimated α diversity (*α_i_*):

$${HC}_{i}={(C\alpha_{i})}^{a} (Eq. 2)$$

where *a* and *C* parameters were to be determined by model fit.

1. Integrating *Eq.1* and *Eq.2*, we can write the formula for estimating habitat capacity (*HC*) based on *PT*×*WA*:

$${HC}_{i}=\left( C\left( {PT}_{i}\times{WT}_{i} \right)^{1.9-1}exp\left( -{PT}_{i}\times{WA}_{i}/4700 \right) \right)^{a} (Eq. 3)$$

**Figure A.** (a) Transformed gamma distribution fitting the relationship between the product of watershed area and annual precipitation and local species richness (*R^2^* = 0.362). The gray crosses were the observed data points, and black line was the best fit by minimizing total error between predicted and observed local species richness. (b) Residual plot of the transformed gamma distribution to estimate alpha diversity with the product of watershed area and annual precipitation. (c) Plot of residuals grouped with sampling seasons with different flow conditions.
