# Supplementary material for: Importance of neutral processes varies in time and space: Evidence from dryland stream ecosystems
Source: PLoS One. 2017 May 9;12(5):e0176949. doi: 10.1371/journal.pone.0176949 (PMC5423606; doi:10.1371/journal.pone.0176949)
Supplement: S3 Appendix — (DOCX) [file pone.0176949.s003.docx]

**Appendix S3.** Method for determining distance matrix for dispersal

First, we extracted two types of distance from the river network: channel distance (i.e., distance along the stream channel) and Euclidean distance (i.e., shortest straight-line distance between the middle points of a pair of streams) (following [1]). To decide which to use, we plotted the relationship between species similarity and pairwise distance, measured with both types of distances. We expected a distance decay relationship [2-3]. We found species similarity decreases with Euclidean distance, but not with stream channel distance (Fig. A). This suggested that the dominant dispersal mechanism for the species in this meta-community was likely dominated by adult areal dispersal, instead of larval aquatic drift in the channel. Using a manipulative experiment in the same system, Bogan & Boersma (2012) [4] showed that about 1/3 of taxa documented from neighboring streams arrived at isolated experimental pools within two weeks via aerial dispersal, lending support to the areal dispersal mechanism.

**Figure A.** The observed pattern of β diversity plotted against pairwise (a) Euclidean distance and (b) channel distance. The grey closed dots in the plots were the actual observed data, and the red open dots were the average Sorensen similarity values for every 5km for the (a) Euclidean distance and every 10km for the (b) channel distance.

**References:**

1. Olden JD, Jackson DA, Peres-Neto PR. Spatial isolation and fish communities in drainage lakes. Oecologia 2001;127: 572-585.
2. Morlon H, Chuyong G, Condit R, Hubbell S, Kenfack D, Thomas D, et al. A general framework for the distance-decay of similarity in ecological communities. Ecol Lett 2008;11: 904–917.
3. Cañedo-Argüelles M, Boersma KS, Bogan MT, Olden JD, Phillipsen I, Schriever TA, et al. Dispersal strength determines meta-community structure in a dendritic riverine network. J Biogeogr 2015;42: 778-790.
4. Bogan MT, Boersma KS. Aerial dispersal of aquatic invertebrates along and away from arid-land streams. Freshw Sci 2012;31: 1131-1144.
