# Supplementary material for: Importance of neutral processes varies in time and space: Evidence from dryland stream ecosystems
Source: PLoS One. 2017 May 9;12(5):e0176949. doi: 10.1371/journal.pone.0176949 (PMC5423606; doi:10.1371/journal.pone.0176949)
Supplement: S4 Appendix — (DOCX) [file pone.0176949.s004.docx]

**Appendix S4**. Comparing model goodness-of-fit at different aggregate levels

The comparison between model results from the model and observed pattern was done at a number of different aggregate levels: (1) cross-season comparison: observed data for a given season were aggregated over three years and compared with results from the model that were aggregated over different years for that same season. And (2) cross-year comparison: observed data were aggregated over three years and compared with results from the model that were also aggregated over the years. To compare model results by year with the three sampling years, we selected years with similar hydrological condition in the model. We first calculated the quantile of annual precipitation of each year (2009 to 2011) within the simulated precipitation series (1000 years after the steady state was reached). For each sampling year, we expanded the range around that year’s quantile until it included 10 simulated years. We used the results from those 10 years to calculate the average patterns and compared them with the patterns of the corresponding sampling year. To aggregate for an annual pattern, we calculated α diversity averaging over *n* sampling visits (*n* =1, 2, 3) within one year as observed α diversity for that year. In the model, mean α diversity across three sampling seasons in a corresponding year was used as α diversity. For the β diversity pattern, observed pairwise data were used to calculated the mean pairwise β diversity of *n* sampling visits in that year. The modeled β diversity was calculated as the mean β across three sampling seasons in the corresponding year.
