# Supplementary material for: Importance of neutral processes varies in time and space: Evidence from dryland stream ecosystems
Source: PLoS One. 2017 May 9;12(5):e0176949. doi: 10.1371/journal.pone.0176949 (PMC5423606; doi:10.1371/journal.pone.0176949)
Supplement: S1 Fig — (DOCX) [file pone.0176949.s005.docx]

**Fig S1.** Observed hydrological regime of inter- and intra-annual variability and hydrological regime used in the model. (a) Observed annual precipitation (1923-2003) and predicted mean annual precipitation using first-order autoregressive model with a gamma residual; (b) normalized discharge from the six USGS gauges within the study area between 2009 and 2011 (the study period), and the modeled durations of the four flow periods (seasons).
