# Supplementary material for: Importance of neutral processes varies in time and space: Evidence from dryland stream ecosystems
Source: PLoS One. 2017 May 9;12(5):e0176949. doi: 10.1371/journal.pone.0176949 (PMC5423606; doi:10.1371/journal.pone.0176949)
Supplement: S1 Table — (DOCX) [file pone.0176949.s006.docx]

**Table S1**. The time of sampling across the 20 sites (streams) between 2009 and 2011. ‘X’ indicated the site was sampled in that given sampling season. ‘SM’: summer; ‘FL’: fall; ‘WT’: winter.

|  | 09SM | 09FL | 10SM | 10FL | 10WT | 11SM | 11FL | 11WT |
| --- | --- | --- | --- | --- | --- | --- | --- | --- |
| Site 1 |  | X | X | X | X | X | X | X |
| Site 2 |  |  |  | X |  |  |  | X |
| Site 3 |  |  | X |  | X | X | X |  |
| Site 4 | X | X | X |  | X | X |  |  |
| Site 5 |  | X | X | X | X | X | X | X |
| Site 6 |  |  | X |  | X |  |  |  |
| Site 7 |  | X |  |  | X |  |  |  |
| Site 8 |  |  |  |  | X |  |  |  |
| Site 9 |  |  |  | X | X | X |  |  |
| Site 10 |  |  |  |  | X | X |  |  |
| Site 11 | X | X | X | X | X | X | X | X |
| Site 12 |  | X |  |  |  |  |  |  |
| Site 13 | X |  |  |  |  |  |  |  |
| Site 14 |  | X |  |  |  |  |  |  |
| Site 15 |  |  |  |  | X |  |  |  |
| Site 16 |  |  | X |  | X |  |  |  |
| Site 17 |  |  |  |  | X |  | X |  |
| Site 18 |  | X |  | X | X |  | X | X |
| Site 19 |  |  |  |  |  |  |  | X |
| Site 20 |  | X | X | X | X | X | X | X |
