# Supplementary material for: Importance of neutral processes varies in time and space: Evidence from dryland stream ecosystems
Source: PLoS One. 2017 May 9;12(5):e0176949. doi: 10.1371/journal.pone.0176949 (PMC5423606; doi:10.1371/journal.pone.0176949)
Supplement: S2 Table — (DOCX) [file pone.0176949.s007.docx]

**Table S2**. List of parameters in the model, their ecological interpretations, and their values used in the best-fit models

| Parameters | Meaning | Value |
| --- | --- | --- |
| *a* | Exponent in the power-law relationship to convert species richness to habitat capacity | 1.05 |
| *C* | Constant in estimating habitat capacity from PT×WA | 6.249 |
| *p* | Dispersal parameter in the 2Dt kernels | 0.40 |
| *l_0_* | Dispersal parameter in the 2Dt kernels; *l_0_* is the distance where, after the dispersal, the ratio between its offspring and those at the origin location is 2^-(1+^*^p^*^)^ | 350 |
| *v* | Speciation rate | 0.0002 |
| *d* | Mortality rate | 0.10 |
| *r* | Recolonization rate | 0.60 |
