# Supplementary material for: Importance of neutral processes varies in time and space: Evidence from dryland stream ecosystems
Source: PLoS One. 2017 May 9;12(5):e0176949. doi: 10.1371/journal.pone.0176949 (PMC5423606; doi:10.1371/journal.pone.0176949)
Supplement: S3 Table — (DOCX) [file pone.0176949.s008.docx]

**Table S3.** Mean local species richness (and its standard deviation in parenthesis) across sampled sites in each sampling season in perrenial streams and non-perrenial streams.

|  | 09SM | 10SM | 11SM | 10WT | 11WT | 09FL | 10FL | 11FL |
| --- | --- | --- | --- | --- | --- | --- | --- | --- |
| Perennial streams | 26.7 (9.0) | 40.2 (15.5) | 49.8 (18.5) | 44.0 (21.8) | 62.7 (17.4) | 57.1 (33.7) | 62.4 (20.9) | 64.0 (21.2) |
| Non-perennial streams | NA | 25.3 (10.3) | 40.0 (1.7) | 18.4 (11.9) | 12.0 (NA) | 23.0 (NA) | 19.0 (22.6) | 64.0 (NA) |
